# Frequency and Evolution of Azole Resistance in *Aspergillus fumigatus* Associated with Treatment Failure^1^

**DOI:** 10.3201/eid1507.090043

**Published:** 2009-07

**Authors:** Susan J. Howard, Dasa Cerar, Michael J. Anderson, Ahmed Albarrag, Matthew C. Fisher, Alessandro C. Pasqualotto, Michel Laverdiere, Maiken C. Arendrup, David S. Perlin, David W. Denning

**Affiliations:** Regional Mycology Laboratory, Manchester, UK (S.J. Howard, D.W. Denning); University of Manchester, Manchester Academic Health Science Centre, Manchester (S.J. Howard, M.J. Anderson, A. Albarrag, D.W. Denning); University Medical Centre, Ljubljana, Slovenia (D. Cerar); Imperial College, London, UK (M.C. Fisher); Universidade Federal do Rio Grande do Sul, Porto Alegre, Brazil (A.C. Pasqualotto); Hôpital Maisonneuve-Rosemont, Montreal, Québec, Canada (M. Laverdiere); Statens Serum Institut, Copenhagen, Denmark (M.C. Ardendrup); Public Health Research Institute, Newark, NJ, USA (D.S. Perlin)

**Keywords:** Aspergillus fumigatus, azoles, antifungal drug resistance, sterol 14-alpha demethylase CYP51A, Aspergillus, population genetics, microsatellites, research

## Abstract

Azoles are the mainstay of oral therapy for aspergillosis. Azole resistance in *Aspergillus* has been reported infrequently. The first resistant isolate was detected in 1999 in Manchester, UK. In a clinical collection of 519 *A. fumigatus* isolates, the frequency of itraconazole resistance was 5%, a significant increase since 2004 (p<0.001). Of the 34 itraconazole-resistant isolates we studied, 65% (22) were cross-resistant to voriconazole and 74% (25) were cross-resistant to posaconazole. Thirteen of 14 evaluable patients in our study had prior azole exposure; 8 infections failed therapy (progressed), and 5 failed to improve (remained stable). Eighteen amino acid alterations were found in the target enzyme, Cyp51A, 4 of which were novel. A population genetic analysis of microsatellites showed the existence of resistant mutants that evolved from originally susceptible strains, different *cyp51A* mutations in the same strain, and microalterations in microsatellite repeat number. Azole resistance in *A. fumigatus* is an emerging problem and may develop during azole therapy.

## Abstract 2

An increase in the frequency of azole-resistant *Aspergillus fumigatus* has emerged.

Invasive aspergillosis in immunosuppressed patients is difficult to diagnose, is problematic to treat, and results in a high mortality rate (*1*). Chronic and allergic pulmonary and sinus aspergillosis are increasingly recognized in numerous clinical settings. Treatment with itraconazole, voriconazole, and, recently, posaconazole is the backbone of therapy for these conditions because azoles are the only licensed class of oral drugs for treatment of aspergillosis (*2**,**3*). Amphotericin B and caspofungin are licensed intravenous agents for invasive aspergillosis but have limited utility for chronic and allergic aspergillosis.

Itraconazole resistance in *Aspergillus* spp. was first reported in 1997 in 3 clinical isolates obtained from California in the late 1980s (*4*); since then, only a few clinical cases have been published (*5**–**9*). The emergence of itraconazole resistance alone is of concern, but widespread azole cross-resistance would be devastating because oral treatment would not be effective.

The primary mechanism of resistance described for *A. fumigatus* clinical isolates is mutation in the target protein. The *cyp51A* gene encodes the target of azoles, lanosterol 14α-demethylase, and this enzyme catalyzes a step in the biosynthetic pathway of ergosterol (an essential cell membrane component of filamentous fungi). Mutations in the open reading frame of the *cyp51A* gene can result in structural alterations to the enzyme, which in turn may inhibit binding of drugs. Mutational hotspots confirmed to cause resistance have been characterized in the gene at codons 54 (*6**,**10**–**13*), 220 (*6**,**14**,**15*), and 98 (*16**–**18*). Other mutations in the *cyp51A* gene have been reported, and additional resistance mechanisms have been postulated (*11**,**19**,**20*). The environmental or antifungal pressures driving azole resistance are unclear because few clinical azole-resistant *Aspergillus* strains have been studied in any detail; many reports simply describe individual patient cases. In this study, we investigated the frequency of *A. fumigatus* itraconazole resistance in a referral laboratory collection, defined the resulting azole cross-resistance pattern, identified mutations in the *cyp51A* gene, and investigated any epidemiologic links between resistant isolates.

## Materials and Methods

### Isolates

Isolates deposited in the Regional Mycology Laboratory Manchester (RMLM) culture collection (between 1992 and 2007) were identified as *A. fumigatus* by macro- and micromorphologic characteristics. All isolates were screened for growth at 50^o^C, thus confirming *A*. *fumigatus* and excluding *A*. *lentulus*. Aspergilli were subcultured onto Sabouraud glucose agar (Oxoid, Basingstoke, UK) for 48 h at 37°C. Thirty-four azole-resistant and 5 susceptible isolates from 17 patients were studied from the RMLM collection (prefixed F); 36 isolates were respiratory specimens, 1 was cerebral, and 2 were from unknown sites. In addition, 18 azole-resistant isolates from a single aspergilloma case-patient (prefixed A, patient 3) collected at autopsy were also investigated.

### Patients

Pertinent details from patients were extracted from the clinical records. All but 6 were under the care of 1 investigator (D.W.D.). Information was collected on underlying disease(s), type of aspergillosis, antifungal treatment, azole plasma levels, and characteristics of therapeutic failure.

### Susceptibility Testing

Susceptibilities were determined by a modified European Committee on Antimicrobial Susceptibility Testing (EUCAST) method (*21*). The modification was a lower final inoculum concentration (0.5 × 10^5^ as opposed to 1–2.5 × 10^5^ CFU/mL). Isolates were tested at a final drug concentration range of 8, 4, 2, 1, 0.5, 0.25, 0.125, 0.06, 0.03, 0.015 mg/L against itraconazole (Research Diagnostics Inc, Concord, MA, USA), voriconazole (Pfizer Ltd, Sandwich, UK), posaconazole (Schering-Plough, Kenilworth, NJ, USA), and amphotericin B (Sigma, Poole, UK). RPMI-1640 (Sigma) was supplemented to 2% glucose (Sigma). Inocula were prepared in phosphate-buffered saline with 0.05% Tween 80 (Sigma); *Aspergillus* spores were counted on a hemacytometer and adjusted to a final concentration of 5 × 10^4^ CFU/mL. Inocula were loaded into flat-bottomed microtiter plates (Costar Corning, Lowell, MA, USA) and incubated at 37^o^C for 48 h. A no-growth end point was determined by eye. MIC testing was performed on RMLM isolates in triplicate, and a consensus mean was derived (median or mode). Susceptibilities of the aspergilloma isolates were determined once, except for 6 that were tested 3 times. Values of >8 mg/L were classed as 16.

Clinical or epidemiologic breakpoints/cutoffs have not been declared by the Clinical and Laboratory Standards Institute (CLSI) or EUCAST for azoles and *Aspergillus* spp. However, proposed epidemiologic cutoff values have been mooted for the latter (*22*), and we have recently proposed clinical breakpoints (*23*). Cutoffs used in this study were itraconazole and voriconazole >2 mg/L and posaconazole >0.5 mg/L (we have not defined an intermediate zone of susceptibility).

### Sequencing

DNA was extracted by using commercially available kits (FastDNA Kit, Q-biogene, Cambridge, UK; Ultraclean Soil DNA Isolation Kit, MO BIO Laboratories Inc., Cambridge; and DNeasy plant tissue kit, QIAGEN, Crawley, UK). The entire coding region of the *cyp51A* gene was amplified as previously described (*7*), except 3 mmol/L MgCl_2_ was used and both strands were sequenced using 8 primers (*7*). Twelve of the aspergilloma (A) isolates were sequenced with only 1 primer, covering the region of interest in this case. Sequences were aligned against the sequence from an azole-susceptible strain (GenBank accession no. AF338659), and mismatches were identified by using AlignX (VectorNTI; Invitrogen, Paisley, UK). Mutations were confirmed by repeating the PCR and sequencing both strands by using the closest 2 primers. Isolates with an alteration in the *cyp51A* gene at codon 98 were also investigated for promoter modifications by sequencing this region (*17*). GenBank accession numbers for the *cyp51A* sequences determined in this study are EU807919−EU807922 and FJ548859−FJ548890.

### Microsatellite Typing

Six microsatellite loci (3A, 3B, 3C, 4A, 4B, 4C) were amplified as previously described (*24*). Initially some amplicons were sequenced, whereas later ones were sized by using capillary electrophoresis on an ABI PRISM 3130×l Genetic Analyzer (Applied Biosystems, Warrington, UK). Electrophoresis data were analyzed by using Peak Scanner Software version 1.0 (Applied Biosystems); amplicon sizes were adjusted by using a correction factor derived from sequenced alleles to determine the actual sizes of alleles (*25*). Concatenated multilocus microsatellite genotypes were created for each isolate and used to generate allele-sharing genetic distance matrices, *D*_AS_. Here, *D*_AS_ = 1 – (the total number of shared alleles at all loci / *n*), where *n* is the total number of loci compared (*26*). Subsequently, phylogenetic comparisons using 5 of the loci (not 3B) were performed with the software PAUP* 4.0 (www.paup.csit.fsu.edu) by using the neighbor-joining algorithm with the minimum-evolution option active. The strength of support for relationships was assessed by using 1,000 bootstrap resamples of the dataset.

## Results

### Susceptibility

The susceptibility of 519 *A. fumigatus* RMLM culture collection isolates was determined. All isolates were tested for susceptibility against itraconazole and amphotericin B; 456 and 118 isolates were also tested against voriconazole and posaconazole, respectively. Subsequently, all itraconazole-resistant isolates were tested against voriconazole and posaconazole. Geometric means, ranges, MIC_50_ (median MIC), and MIC_90_ (90% of the isolates tested had a MIC at or below this level) values are shown in Table 1. Amphotericin B susceptibility was retained in the 34 itraconazole-resistant isolates tested. Of these, 65% (22) were cross-resistant to voriconazole and 74% (25) were cross-resistant to posaconazole. We did not identify any isolates that were resistant to voriconazole or posaconazole while remaining susceptible to itraconazole.

**Table 1:** MICs for 519 *Aspergillus fumigatus* isolates from RMLM culture collection, 1992–2007*

Isolate group (no. isolates)	Susceptibility results, mg/L										
	Itraconazole			Voriconazole			Posaconazole			Amphotericin B	
	GM (range)	MIC_50_/ MIC_90_		GM (range)	MIC_50_/ MIC_90_		GM (range)	MIC_50_/ MIC_90_		GM (range)	MIC_50_/ MIC_90_
RMLM collection (519), 1992–2007	0.46 (<0.015–>8)	0.25/2		0.92 (0.125–>8)	1/2		0.22 (0.03–>8)	0.125/2		0.34 (0.06–2)	0.25/1
Azole resistant (34)	16.0 (>8)	>8/>8		3.69 (0.125–>8)	4/>8		1.70 (0.125–>8)	1/>8		0.22† (0.06–0.5)	0.25/0.5
Percentage resistant	100%			65%			74%			0%	
Aspergilloma (18)	16.0 (>8)	>8/>8		2.16 (0.5–4.0)	4/4		1.92 (0.125–>8)	1/>8		0.10‡ (0.06–0.125)	0.125/ 0.125

*RMLM, Regional Mycology Laboratory Manchester; GM, geometric mean. Values >8 mg/L were classed as 16 mg/L for GM analysis. See also the Appendix Table. †n = 28. ‡n = 6.

**Figure 1:**
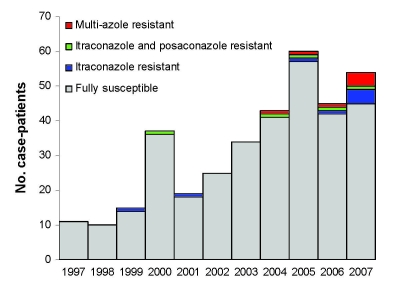
Azole resistance in clinical *Aspergillus fumigatus* isolates received in the Regional Mycology Laboratory Manchester, UK, 1997–2007. Overall azole resistance for each year is shown above each column as a percentage. Data do not include sequential isolates from the same patient.

Five percent of 400 isolates were resistant to itraconazole (when duplicate isolates from the same patient with similar susceptibility profiles were removed from the analysis). The overall frequency of itraconazole resistance in this collection (with repeat specimens included) was 7% (n = 519). The first case of azole resistance in this collection was seen in 1999. The frequency of resistance since 2004 (8%) has increased significantly (Fisher exact test, p<0.001), compared with the period prior to 2004 (Figure 1).

### Azole Exposure in Patients with Azole-Resistant Isolates and Response to Therapy

Of the 17 patients identified for respective review, limited data were available for 3 patients. Of the remaining 14 patients with antifungal data (Table 2), azole exposure of 1–30 months before the identification of the first resistant isolate was evident for all except patient 7. Thirteen patients received itraconazole as initial therapy, and 12 of these were evaluable. Infections failed to respond to therapy in 7 itraconazole-treated patients (i.e., their disease progressed), although 3 patients appeared to improve before their conditions began to deteriorate. Infections failed to improve with azole therapy in 5 patients (i.e, their disease remained stable), and no patient had a sustained response to therapy. Nine of the 12 patients had at least 1 therapeutic concentration of itraconazole (>5.0 mg/L) documented at steady state during their treatment course (online expanded version of Table 2, available from www.cdc.gov/EID/content/15/7/1068-T2.htm). The infections in patient 1 (treated with voriconazole only for 18 months) failed therapy, and the 1 isolate identified had MICs of >8 mg/L for both itraconazole and voriconazole.

**Table 2:** Clinical information for 17 patients with azole resistant *Aspergillus fumigatus* infections*

Patient no.	Age, y/sex	City	No. isolates	*Aspergillus* disease	Other diseases, y	Daily dose, duration	Serum azole levels, mg/L†	Outcome	Survival
1	50/F	Cambridge, UK	1	CCPA with aspergilloma	Breast cancer, 1990; *M. malmoense* pulmonary tuberculosis, 1999 and 2005	Vori 200–400 mg, 18 mo	ND	Clinical and radiological failure	Alive
2	21/F	Copenhagen, Denmark	1	ABPA	CF, concomitant bacterial colonization with *Staphylococcus aureus* and *Achromobacter*	Itra 200 mg, 14 mo (plus previous courses)	ND	Unknown	Alive
3	40/F	Manchester, UK	2‡	CCPA with aspergilloma, then CFPA	Pulmonary TB with residual bilateral UL scarring and LUL cavity, 1986; smoke inhalation, 1989	Itra 400 mg, 90 mo	15.0–26.0§	Clinical failure	Died
4	72/M	Manchester, UK	3	CCPA with aspergilloma	COPD, squamous cell carcinoma with LUL segmentectomy, 1992	Itra 400 mg, >2 mo	2.9–11.3	No improvement	Died
5	43/M	Montreal, Quebec, Canada	2	Cerebral aspergillosis, Nov 1998	AML-M2, 1997; RUL lobectomy, 1997; AlloHSCT, 1998; GVHD	Itra 400 mg, 4 mo	ND	Regression of cerebral abscess, then IPA with respiratory failure	Died
6	60/M	Manchester, UK	2	CCPA with aspergilloma	COPD, *M. szulgai* pulmonary infection, 2003; celiac disease	Itra 200–400 mg, 1 mo	<0.8 (200 mg), 5.3–7.7 (400 mg)	Clinical failure	Died
7	77/M	Manchester, UK	1	Acute invasive pulmonary	COPD, possible bronchiectasis	Itra 600–400 mg, 1 mo; vori 400 mg, 12 d	17.0–21.0 (itra)	No improvement; switched to vori, developed toxicity	Died, without IPA
8	46/F	Northampton, UK	2	ABPA	Bronchiectasis, asthma, AVR, hypermobility syndrome*, M. xenopi* pulmonary infection, 2007	Itra 200–400 mg, 9 mo	0.0–5.2	Initial improvement, then failure	Alive
9	46/M	Liverpool, UK	12	CCPA with bilateral aspergillomas, CFPA	Pulmonary sarcoidosis, 1988	Itra 200–400 mg, 30 mo	0.9–10.3	Clinical failure	Died
10	41/F	Manchester, UK	2	*Aspergillus* bronchitis	Bronchiectasis, onychomycosis, 2007; α-1-antitrypsin deficiency	Itra 400 mg pulse, 3 mo	ND	Itra resistance identified, so treated with posa	Alive
11	62/F	Manchester, UK	2	CCPA with aspergilloma	RUL pneumonia, 2002	Itra 400 mg, 1.5 mo	20.0–>25.6	No improvement	Alive
12	29/F	Manchester, UK (Malawi origin)	1	CCPA with 2 aspergillomas	Pulmonary TB, 1995; HIV positive, HAART	Itra 400 mg, 18 mo	2.5–8.4	Improvement then progression	Alive
13	64/M	Preston, UK	4	CCPA with aspergilloma	COPD, bronchiectasis, *M. avium* pulmonary infection, 2002 and 2006	Itra 600 mg, 10 mo	2.6–4.5	Progression	Alive
14	42/M	Birkenhead, UK	1	CCPA with LUL aspergilloma	Sarcoidosis, COPD, celiac disease; aspergilloma removed as part of left lung transplant, 2007¶	Itra 400 mg, 11 mo	13.8–17.8	Unchanged, switched to vori	Unknown
15	68/F	Wirral, UK	1	Sputum isolate	Cardiac transplant for congestive cardiomyopathy, 1999; chronic cough; 2007; polymyalgia rheumatica, hiatal hernia, oesophagitis	Not documented	NA	Not assessable	Alive
16	12/F	Liverpool, UK	1	Sputum isolate	Unknown	Unknown	Unknown	Unknown	Unknown
17	43/M	Manchester, UK	1	Sputum isolate	Unknown	Unknown	Unknown	Unknown	Unknown

*CCPA , chronic cavitary pulmonary aspergillosis; *M.*, *Mycobacterium*; vori, voriconazole; ND, not determined; ABPA, allergic bronchopulmonary aspergillosis; CF, cystic fibrosis; itra, itraconazole; CFPA, chronic fibrosing pulmonary aspergillosis; TB, tuberculosis; UL, upper lobe; LUL, left upper lobe; COPD, chronic obstructive pulmonary disease; AML, acute myeloid leukemia; RUL, right upper lobe; AlloHSCT, allogeneic haematopoietic stem cell transplant; GVHD, graft versus host disease; IPA, invasive pulmonary aspergillosis; AVR, aortic valve replacement; posa, posaconazole; HAART, highly active antiretroviral therapy. †Determined by bioassay (target range 5–15 mg/L). ‡Plus aspergilloma isolates studied, taken at autopsy. §Received a generic formulation of itra, resulting in lower concentrations (i.e., 4.6 mg/L) and then probably was noncompliant at end of treatment period. ¶Successfully completed with vori treatment.

Of the 14 patients with available data, 2 had invasive disease; 9 had chronic disease with >1 aspergillomas; 2 had allergic bronchopulmonary aspergillosis; and 1 had *Aspergillus* bronchitis. At least 5 of the patients died of progressive infection, despite alternative therapies for some.

### Mutations in the *cyp51A* Gene

A summary of Cyp51A amino acid substitutions and azole cross-resistance patterns identified in 34 resistant isolates from our clinical culture collection is shown in Table 3 and listed by line in the Appendix Table. The sequences of all 5 azole-susceptible isolates examined were identical to that of a previously published *cyp51A* gene sequence from an azole-susceptible isolate (AF338659). No *cyp51A* mutations were found in 3 itraconazole-resistant isolates (from 2 patients). In addition to the L98H substitution, 2 isolates from 2 patients had a 34-bp sequence that was duplicated in the promoter region (*16**,**17*) of the *cyp51A* gene. One isolate had 2 amino acid substitutions, H147Y and G448S. Three isolates from 2 patients had the same 6 mutations, 3 nonsynonymous ones (F46Y, M172V, E427K), along with 3 synonymous (silent) alterations at codons 89, 358, and 454 (data not shown), and an isolate from a third patient had additional mutations (N248T, D255E) as well as these 6. Four novel mutations were found (H147Y, P216L, Y431C, and G434C). The isolate bearing the P216L mutation was resistant to itraconazole and posaconazole, whereas the isolates with Y431C and G434C showed pan-azole resistance phenotypes.

**Table 3:** Cyp51A amino acid substitutions and associated cross-resistance patterns in azole-resistant RMLM *Aspergillus fumigatus* isolates*

Cyp51A codon	No. patients	No. isolates	Amino acid substitutions	MIC, mg/L†		
				Itraconazole	Voriconazole	Posaconazole
F46‡	3	4‡	Y	>8	2–4	0.125–0.5
G54	4	5	E, R, V	>8	0.125–1	1–>8
L98+TR	2	2	H	>8	8	1–2
G138	1	10	C	>8	8–>8	2–>8
H147§	1	1§	Y	>8	>8	0.5
M172‡	3	4‡	V	>8	2–4	0.125–0.5
P216	1	1	L	>8	1	1
M220	3	4	K, T	>8	1–4	0.5–>8
N248‡	1	1	T	>8	2	0.25
D255‡	1	1	E	>8	2	0.25
E427‡	4	5‡	G, K	>8	2–4	0.125–0.5
Y431	1	1	C	>8	4	1
G434	1	1	C	>8	4	1
G448	2	2	S	>8	>8	0.5–1
No substitutions	2	3	NA	>8	2–8	0.25–1

*RMLM, Regional Mycology Laboratory Manchester; TR, tandem repeat in *cyp51A* promoter; NA, not applicable. Synonymous mutations not shown. Some mutations are associated with resistance but may not be causal (see text). †Putative cut-off values for resistance are itraconazole and voriconazole >2 mg/L and posaconazole >0.5 mg/L. ‡F46Y found with M172V and E427K in 4 isolates along with 3 silent mutations. E427G seen alone in 1 isolate. N248 and D255 found in combination with 46/172/427 in 1 isolate. §Found with G448S in 1 of 2 isolates.

Patient 3 had 2 respiratory samples taken while she was alive, in addition to 18 aspergilloma isolates sampled at autopsy. All isolates were resistant to itraconazole (>8 mg/L), and 1 of 2 different mutations at codon 220 was detected in the *cyp51A* gene. Isolates with a methionine-to-lysine substitution were highly cross-resistant to voriconazole (4 mg/L) and posaconazole (>8 mg/L), whereas those with an alteration to threonine had variable voriconazole (0.5–4 mg/L) and posaconazole (0.125–1 mg/L) MICs.

### Microsatellite Typing

**Figure 2:**
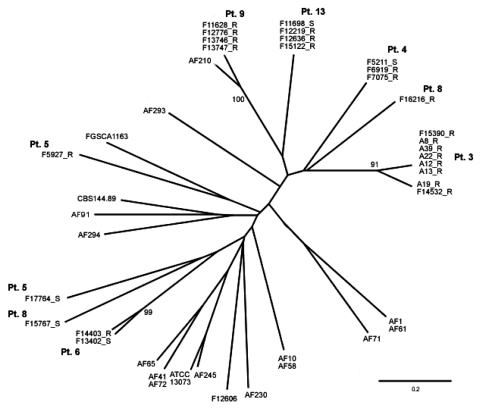
Unrooted phylogenetic tree showing the genetic relationship of isolates from 7 patients.The genetic relationship of these isolates is shown in relation to each other and to 18 other isolates. AF numbers belong to a collection of >200 isolates, held in Manchester, UK. ATCC, American Type Culture Collection; CBS, Centraalbureau voor Schimmelcultures; FGSC, Fungal Genetics Stock Center. Bootstrap values >90 only are shown. Scale bar indicates nucleotide substitutions per site.

The relatedness of isolates obtained from patients 3, 4, 5, 6, 8, 9, and 13 were compared by microsatellite typing (Figure 2). The isolates from 5 patients consisted of a susceptible/resistant pair, whereas an overlapping group of 4 patients had more than 1 *cyp51A* mutation. All isolates were from the lower respiratory tract, except the resistant isolate from patient 5, which was from a cerebral lesion.

Multiple isolates from 5 of 7 patients had identical or nearly identical genotypes. The isolates from 2 of these 5 patients (3 and 6) differed by 1 and 2 trinucleotide repeat units, respectively, at the most polymorphic locus (3A). Three matched sets (isolates pre- and postdevelopment of resistance) were identified, where resistance almost certainly evolved from an originally susceptible strain.

Figure 2 shows an unrooted tree of the phylogenetic relationships, derived from 5 of the 6 microsatellite markers, for the isolates from these 7 patients plus 18 *A. fumigatus* isolate controls. Only bootstrap values >90 are shown. Strains from these 7 patients are distributed among other clinical isolates; statistically supported clustering is not evident. Therefore, none of the azole-resistant isolates have been transmitted from patient to patient, indicating that they have all evolved independently from different original strains. The only statistically supported clades contain isolates that only differ from each other by 1 of the 5 markers.

## Discussion

Itraconazole resistance and azole cross-resistance in *Aspergillus* spp. have been reported infrequently, which suggests that they are infrequent events to date. A contributing factor to this low prevalence has been variability in testing between laboratories. Since the initial report of resistance in isolates collected before the licensure of itraconazole, substantial improvements in susceptibility testing methods that allow confidence in reported azole MICs have been implemented. Recommended methods are now promulgated by the CLSI method M38-A2 (*27*) and EUCAST (*21*), and work is ongoing to establish internationally agreed interpretative cutoffs (*22*) and clinical breakpoints (*23*).

By using such methods, some researchers have documented and published the frequency of itraconazole resistance in clinical *A. fumigatus* isolates (*8**,**28**–**32*); frequency ranged between 2% and 6%. However, most of these studies included fewer isolates (<200) than our study (519) and covered the pre-2004 era. The frequency of itraconazole resistance in our collection before 2004 was 1%; since 2004, however, it has been remarkably high at 8%. The high frequency probably reflects, at least in part, the specialized referral base for patients with chronic and allergic aspergillosis at our center, although there has been no material change in catchment area in the past decade. Referral numbers are rising, however, and susceptibility testing of isolates of patients receiving therapy has been more frequent since 2003.

Another remarkable aspect of this study is the diversity of *cyp51A* mutations. Both previously published and novel alterations were identified in our resistant isolates (Table 3). In contrast, a recent series of 32 itraconazole-resistant isolates from the Netherlands was published; 94% had the same 2 alterations: an L98H-aa substitution in Cyp51A, in combination with a duplication in the promoter region (*32*). This combination of mutations was found in 2 of our isolates from 2 patients.

Several authors have identified hot-spot regions associated with resistance in clinical isolates at codons 54 (*6**,**10**–**13**,**22*), 98 (*16**–**18**,**22**,**33*), and 220 (*6**,**14**,**15**,**22**,**32*) in the *cyp51A* gene. We previously reported an alteration at codon 138 (G138C) in multiple isolates from 1 patient (*7*). A single clinical isolate with a mutation at codon 448 (G to S) has also been previously reported (*34*). In addition, G138R and G448S mutants have been generated in the laboratory and were azole resistant (*35*). Mutations in codons 46, 172, 248, 255, and 427 have been found in azole-susceptible strains by us (A. Albarrag, unpub. data) and others (*22*) and so are not associated with resistance. The resistant isolates with these mutations must therefore have another resistance mechanism. Four novel *cyp51A* mutations, 3 of which were unassociated with any other mutations (in codons 147, 216, 431, and 434), were identified in this series, although their association with resistance remains to be confirmed experimentally. The H147Y substitution is probably unimportant for resistance because it was found with G448S in 1 isolate and the cross-resistance profile of this isolate was identical to an isolate that had only G448S. We did not find any examples of previously reported mutations at codons 297 and 495 (*17**,**32*) or 22, 394, 491, and 440 (*14*) in our collection. Three of our resistant isolates had no mutations in their *cyp51A* gene, indicating the presence of other resistance mechanisms.

The position and type of amino acid substitution within the Cyp51A protein determines the pattern of azole cross-resistance (Table 3), which is consistent with predicted structural properties of the demethylase enzyme and its interaction with chemically different azole drugs (*36*). Resistance to itraconazole is usually associated with a reduction in posaconazole susceptibility, predictably because the 2 drugs are structurally similar; they have variably elevated posaconazole MICs compared with wild type isolates (*22**,**30*). Many of the isolates reported here reflect this MIC shift. Isolates with alterations at codons 98 (including the duplication in the promoter region), 138, 431, and 434 demonstrated cross-resistance to voriconazole and posaconazole. All isolates with substitutions at codons 54 and 216 remained susceptible to voriconazole. Some isolates in this study showed cross-resistance between itraconazole and voriconazole and not posaconazole, unlike the results in previous reports (*22**,**32*). However, this cross-resistance could be because of differing breakpoints; therefore, determination of an internationally agreed cutoff for posaconazole will be necessary to guide clinicians. No difference in amphotericin B MICs was seen in our azole-resistant isolates compared with susceptible ones, although the clinical utility of MIC testing of amphotericin B in *Aspergillus* spp. is suboptimal.

More than 1 azole-resistant *A. fumigatus* isolate was obtained from 6 of the 17 patients described. Microsatellite typing demonstrated that the isolates from each patient had evolved from a single original strain, because they were either identical at 6 markers or differed only in the most polymorphic marker. The resistant isolates from 4 patients had different *cyp51A* mutations. Given that a single colony is picked from the primary isolation plate and referred for susceptibility testing, additional mutants may have been found had multiple colonies been tested. Within this dataset, the chance of 2 isolates being identical by chance alone within a recombining population are infinitesimal given the high allelic variability that we observed (mean number of alleles per locus = 14; p recovering the same multilocus genotype twice ≈14^5^). The existence of susceptible and resistant isolates that are genetically identical from 3 patients, and the phylogeny performed on the multiple-resistant isolates from an additional 4 patients, almost certainly indicates that the evolution of azole resistance has occurred in these patients independently and repeatedly from unrelated strains. The presence of genetically identical isolates with different *cyp51A* codon mutations in 3 patients (and 1 almost identical) suggests that they must have evolved independently from the same original strain, because the resistance mutations are not being accumulated sequentially as has been shown to happen in *Candida albicans* (*37*). The isolates from 2 patients had differing numbers of repeats of microsatellite marker 3A, which is further proof that strains are evolving in the lung. In contrast, Snelders et al. (*32*) suggested that many of their patients were infected with a primary resistant strain from the environment.

The referral base for these isolates includes a specialized clinical service for the management of aspergillosis. Many of our resistant isolates came from this group, in particular from 9 patients with chronic cavitary pulmonary aspergillosis with >1 aspergillomas, which may explain the high frequency of resistance in our center. Because surgery is not an option for most patients with chronic cavitary pulmonary aspergillosis, these patients usually require long-term (if not lifelong) antifungal therapy, under which conditions as we have shown, strains of *A. fumigatus* may evolve resistance. Another contributory explanation could be our systematic application of susceptibility testing of *Aspergillus* spp. isolates in all cases in which treatment is to be given.

In 6 of 10 patients, steady state itraconazole plasma level data were at or above minimum therapeutic levels (i.e., <5 mg/L), as determined by bioassay (*38**,**39*). Low plasma levels of itraconazole were attributable to limited bioavailability in some patients, low doses (i.e., 200 mg daily, the standard UK registered dose), drug interactions in patients with concomitant atypical mycobacterial infection, and use of generic itraconazole (*40*). Low plasma levels of itraconazole, in combination with the high proportion of patients in this study with prior azole exposure (13 out of 14), indicates that resistance primarily emerged during or after azole therapy.

Our observations are of concern on several fronts. We found a sudden rise in the frequency of azole resistance in *A. fumigatus* since 2004, and many isolates showed cross-resistance between all the currently licensed azole options. Clinical data indicate that resistance has occurred during and after azole therapy in all but 1 of these cases. The infections caused by azole-resistant isolates fail therapy or at best do not respond. The molecular epidemiology shows that resistance evolved in infecting strains within the lung, rather than by superinfection with a resistant stain from the environment. Because azoles are the only useful class of oral drugs for aspergillosis (and many other serious filamentous fungal infections), clinical management of these chronically infected cases is therefore problematic. Vigilance is called for to identify azole-resistant aspergilli, and novel classes of oral antifungal would be welcome for those infected with azole-resistant strains.

## Supplementary Material

Appendix Table Isolates, MICs, cyp51A mutations, and molecular similarity, by patient*
