# Supplementary material for: Frequency and Evolution of Azole Resistance in Aspergillus fumigatus Associated with Treatment Failure
Source: Emerg Infect Dis. 2009 Jul;15(7):1068–76. doi: 10.3201/eid1507.090043 (PMC2744247; doi:10.3201/eid1507.090043)
Supplement: Appendix Table — Isolates, MICs, cyp51A mutations, and molecular similarity, by patient* [file 09-0043_appT-s1.pdf]

Appendix Table. Isolates, MICs, *cyp51A* mutations, and molecular similarity, by patient*

Patient no.	Isolate	Susceptibility			Cyp51A amino acid substitutions	Molecular type
		Itraconazole	Voriconazole	Posaconazole		
1	F13619	>8	>8	0.5	H147Y G448S	ND
2	F16134	>8	4	>8	M220K	ND
3	F14532	>8	1	0.5	M220T	Probable same type
3	F15390	>8	4	1	M220T	
3	A8	>8	4	>8	M220K	
3	A12	>8	4	>8	M220K	
3	A13	>8	1	0.25	M220T	
3	A19	>8	1	0.25	M220T	
3	A22	>8	2	1	M220T	
3	A39†	>8	1	0.25	M220T	
4	F5211	0.5	1	0.125	NF	Same type
4	F6919	>8	2	2	M220K	
4	F7075	>8	1	>8	G54E	
5	F17764	0.25	2	0.06	NF	Different type
5	F5927	>8	2	0.125	F46Y M172V E427K	
6	F13402	0.125	0.5	0.06	NF	Probable same type
6	F14403	>8	0.5	>8	G54R	
7	F7763	>8	2	0.25	F46Y M172V N248T D255E E427K	ND
8	F15767	0.25	0.5	0.06	NF	Different type
8	F16216	>8	8	2	L98H+TR	
9	F11628	>8	>8	>8	G138C	Same type
9	F12041	>8	>8	>8	G138C	
9	F12760	>8	8	2	G138C	
9	F12776	>8	4	1	Y431C	
9	F12865	>8	8	2	G138C	
9	F13535	>8	8	2	G138C	
9	F13746	>8	8	2	G138C	
9	F13747	>8	4	1	G434C	
9	F13952	>8	>8	>8	G138C	
9	F14513	>8	>8	>8	G138C	
9	F14811	>8	>8	>8	G138C	
9	F14946	>8	>8	>8	G138C	
10	F16311	>8	8	1	NF	ND
10	F16351	>8	4	0.5	NF	
11	F15651	>8	4	0.25	F46Y M172V E427K	ND
11	F17103	>8	4	0.5	F46Y M172V E427K	
12	F16157	>8	0.5	1	G54V	ND
13	F11698	0.25	0.5	0.03	NF	Same type
13	F12219	>8	0.125	1	G54R	
13	F12636	>8	0.125	1	G54E	
13	F15122	>8	>8	1	G448S	
14	F15861	>8	1	1	P216L	ND
15	F16510	>8	2	0.25	NF	ND
16	F16867	>8	2	0.125	E427G	ND
17	F17294	>8	8	1	L98H+TR	ND

*ND, not determined; NF, not found; TR, tandem repeat in *cyp51A* promoter region.

†The *cyp51A* gene was partially sequenced for an additional 12 isolates from this patient (data not shown).
